# Green waste compost as an amendment during induced phytoextraction of mercury-contaminated soil

**DOI:** 10.1007/s11356-014-3601-5

**Published:** 2014-09-24

**Authors:** Beata Smolinska

**Affiliations:** Institute of General Food Chemistry, Department of Biotechnology and Food Sciences, Lodz University of Technology, 4/10 Stefanowskiego Street, 90-924 Lodz, Poland

**Keywords:** Assisted phytoextraction, Mercury, *Lepidium sativum* L, Compost

## Abstract

Phytoextraction of mercury-contaminated soils is a new strategy that consists of using the higher plants to make the soil contaminant nontoxic. The main problem that occurs during the process is the low solubility and bioavailability of mercury in soil. Therefore, some soil amendments can be used to increase the efficiency of the Hg phytoextraction process. The aim of the investigation was to use the commercial compost from municipal green wastes to increase the efficiency of phytoextraction of mercury-contaminated soil by *Lepidium sativum* L. plants and determine the leaching of Hg after compost amendment. The result of the study showed that Hg can be accumulated by *L. sativum* L. The application of compost increased both the accumulation by whole plant and translocation of Hg to shoots. Compost did not affect the plant biomass and its biometric parameters. Application of compost to the soil decreased the leaching of mercury in both acidic and neutral solutions regardless of growing medium composition and time of analysis. Due to Hg accumulation and translocation as well as its potential leaching in acidic and neutral solution, compost can be recommended as a soil amendment during the phytoextraction of mercury-contaminated soil.

## Introduction

Mercury is a highly toxic contaminant that can be found in the environment. Its toxicity for plants is influenced by Hg species present in soil, which is a function of many soil factors that include physicochemical soil properties, such as soil pH, texture, organic matter, clay contents, cation exchange capacity, and redox potential. Moreover, initial Hg concentration in soil solution can also affect its toxicity and availability to plants (Planquart et al. 1999; Businelli et al. 2009). There are two sources of mercury in the environment—natural and anthropogenic. Although the concentration of Hg that enters the environment from the natural source has remained the same over the years, the increasing concentration of this element is still observed. Coal burning, waste combustion, mining, and industrial practices are the main anthropogenic sources. Mercury enters the environment mostly in gaseous form. Moreover in this form, Hg can be transported at high distances and deposited into the soil contributing to its contamination. In soil, mercury can be absorbed by microorganisms and plants which results in transferring this element to the food chain. Therefore, there is an actual danger for all living organisms including human (Alloway 1995).

There are a lot of different methods useful for cleaning soil contaminated by Hg. The application of physical or chemical methods, like washing the soil with halogenated substances and heating it in high temperatures, chemical extraction, or electro remediation (Hempel and Thoeming 1999; Renneberg and Dudas 2001; Thoming et al. 2000) is mostly limited by the economic point of view. The possibility exists in biological techniques, like phytoextraction. Phytoextraction consists of using the higher plants to take up pollutants from contaminated medium (soil or water), transport, and accumulate them into the shoots of plants. The pollutants can then be removed by harvesting the aboveground tissues. There are two strategies of phytoextraction—natural or chelate-induced process. The natural phytoextraction consists of using plants that can accumulate extraordinary levels of contaminants in their tissues. These plants are called hyperaccumulators. The second strategy—chelate-induced phytoextraction, consists of using simple higher plants and soil amendments to increase the accumulation by plants (Evangelou et al. 2007).

There are different chemical compounds that can be used as chelators during mercury phytoextraction process. This group of compounds include substances that show chemical affinity to mercury and/or can form stable complex compounds with mercury ions, like cysteine (Cyst), potassium iodide (KI), or thiosulphate ((NH_4_)_2_S_2_O_3_) (Lomonte et al. 2011). The researches have shown (Wang and Greger 2006; Lomonte et al. 2011) that application of the mentioned substances to the soil increases the Hg solubility in soil solution and at the same time increases the bioavailability of this element. Moreover, the application of these chemical chelators showed positive effect on the efficiency of Hg phytoextraction process.

Despite the undoubtedly advantages of chelate-induced phytoextraction process, using chemical chelators can increase the risk of spreading pollutants from soil to other environmental components, like groundwater. All the investigated substances in the field of their potential in assisting phytoextraction increased the leaching of Hg to groundwater. Therefore, their application in the natural environmental conditions should be carefully considered. Concerning all aspects of chelate-induced phytoextraction, the researches are still focused on finding the economically reasonable substances that can increase the efficiency of Hg phytoextraction without causing the risk of leaching. The substance that has that potential is compost. Compost is a natural substance that is characterized by high content of organic matter as well as high concentrations of macro and micronutrients. Municipal green waste (MGW) compost is formatted from the leaves, grasses, branches, and other green wastes. This kind of product meets the safe and quality standards and can be used as a fertilizer during the cultivation of the consumption plants species. Moreover, compost can be use as an amendment during assisted phytoextraction of heavy metal-contaminated soils. Its role consists on increasing metal availability by the formation of soluble metal-organic complexes thus increasing plant uptake efficiency (Businelli et al. 2009; Zheljazkov and Warman 2004).

Several studies have attributed increased metal solubility, mobility, and bioavailability of soils treated with organic amendments during soil reclamation (Karami et al. 2011; Beesley and Dickinson 2010; Beesley et al. 2010). These researches have demonstrated that application of different composts to the soil increased the formation of soluble organo-metallic complexes with dissolved organic carbon (Brunetti et al. 2012). However, the investigations were performed for different heavy metals, like Cr, Cu, Pb, Zn, but not mercury, that were found in both soil and compost (Zhao et al. 2013; Fornes et al. 2009). Therefore, the aim of the present investigation was to determine the efficiency of MGW compost application on the Hg phytoextraction process by *Lepidium sativum* L. plants. The study also includes the determination of leaching the mercury after phytoextraction enhanced by MGW compost.

## Materials and methods

### Soil and compost

Soil was collected from the surface layer of 0–30 cm in Lodz, Poland. It was a sandy loam soil with density of 1.2 ± 0.1 g cm^−3^. The soil samples were prepared for further analysis according to ISO 11464:1999. After being air-dried, the soil was passed through a 2-mm nylon mesh. The following soil properties were characterized: pH according to ISO 10390:2005, organic matter according to ISO 14235:2003, total nitrogen according to ISO 11261:2002, and available phosphorous according to Polish Standard PN-P-04023:1996. The pseudototal content of heavy metals was determined in triplicate by acid mineralization in aqua regia according to ISO 11466:2002. Concentrations of Pb, Cu, and Zn were measured by flame atomic absorption spectrometry (F-AAS) (ISO 11047:2001). Level of Hg was determined in accordance with ISO 16772:2009 by cold vapor atomic adsorption spectrometry (CVAAS). In all determinations of atomic absorption, J.T. Baker standards were used. Extraction of blanks was included in each batch of 10 samples. The accuracy of the method was confirmed by the analysis of reference standard 0217-CM-7001-04. The recovery percentages were as follow: 108 % for Pb, 99 % for Zn, 96 % for Cu, and 97 % for Hg.

MGW compost used in the study originated from the Institute of Municipal Services in Lodz, placed at 70/72 Sanitariuszek Street, Lodz, Poland. This commercially available compost *Prochniaczek* was mainly obtained from branches, leaves, and grasses collected as wastes in Lodz city. The compost was air-dried and sieved through a 2-mm nylon mesh for chemical analysis and other investigations. The compost was characterized with the following European Standards for soil improvers and growing media: pH according to EN 13027:2011, organic matter according to EN 13039:2007, and total nitrogen according to 13654 1:2001. Chosen heavy metal contents as well as mercury concentration were measured following the method described above for soil. In further description in the investigation, term compost will be used replaceable of MGW compost.

### Experimental design

#### Characteristic of the growing medium

Mercury(II) chloride (HgCl_2_) in concentration 10 and 100 mg kg^−1^ soil dry weight was used as a contaminant. Mercury solution was uniformly mixed with air-dried soil and left for 24 h. The greenhouse experiments were conducted at growing media consisted of:

- uncontaminated natural soil (blank soil sample)
- soil samples contaminated by Hg in concentrations 10 and 100 mg kg^−1^ dry weight (two control samples)
- soil and compost in soil/compost ratio 2/1, 3/1, and 4/1 contaminated by Hg in concentrations 10 and 100 mg kg^−1^ dry weight separately (six samples)

The soil and compost were mixed and made the homogeneous growing medium for plant cultivation. Each of the variant of experiment runs in triplicate.

#### Phytoextraction

The phytoextraction was carried out in nine variants of soil/growing medium, listed in “Characteristic of the growing medium” section. The experiment was set up with plastic pots in a greenhouse with day/night system of 22/19 °C and photoperiod of 14 h. Ten grams of *L. sativum* L. seeds per 1 kg of growing medium was put into the plastic pots. The plants were watered to keep the growing medium humidity of about 35 % during the experiment period. The cultivation was provided within 10 days from sowing.

To evaluate the Hg concentration in *L. sativum* L., the examined plants were harvested, washed to remove soil particles, and separated the below and above parts (roots and shoots, respectively). The roots and shoots were weighed separately to determine the biomass and air-dried. The dried plant samples were ground to a powder and in this form, subjected to mineralization according to Cavallini et al. (1999). The concentration of Hg in plant samples were determined using CVAAS method. Extraction of blanks was included in each batch of 10 samples. The accuracy of the Hg determination in plant samples was confirmed by the analysis of standard ERM-CD281. The recovery of the method was 98 %.

The biometric parameters of plants which included the length of roots and shoots as well as the plant biomass were determined according to Carrasco-Gil et al. (2012).

Bioconcentration factor (BCF) was calculated as a ratio of element concentration in plant shoots to total element concentration in soil/growing medium according to Perez-Sanz et al. (2012).

#### Leaching of Hg

The leaching experiments were provided in two water solutions—at pH = 7 (distilled water) and pH = 5 (water solution of HNO_3_) after plant cultivation. Fifteen grams of each growing medium (soils as control samples and soil/compost ratio 2/1, 3/1, and 4/1) was put separately in the round-bottomed flask, and 150 mL of water solution was added. The flask was shaken for 1 h in room temperature (20 °C). After shaking, the sample was left for 24 h at room temperature and then filtered. The extract was acidified by the concentrated nitric (V) acid, and the concentration of Hg in the extract was determined. Each of the variant of analysis was done in triplicate, in regular time periods: 1, 5, 10, 15, and 20 days after phytoextraction was finished. The concentration of Hg in solutions was determined by CVAAS after microwave mineralization.

### Statistical analysis

The results reported in this study were means of the three replicates with standard deviation values. Statistical analysis was carried out on all results using ANOVA Excel Data Analysis. Analysis of variance was performed to determine the significance of differences between the pairs of means in different variants of the conducted experiment. The differences were statistically significant when *P* value was less than 0.05. Multiple range test, based on Fisher’s last significant (LSD) procedure, was done to find out which means are significantly different from others.

## Results and discussion

### Characteristic of soil and compost

Table 1 shows the properties of soil and compost used in the present study. The investigations concerning the physicochemical soil properties were provided on natural, unpolluted soil which constituted the blank sample. Determination of soil pH enables to include the soil to slight acidic according to Soil Survey Division Staff (1993). Concentration of organic carbon, total nitrogen, and available phosphorous was rather low but sufficient for plant cultivation. Soil was uncontaminated by mercury—the amount of this element was below the level of detection (<0.005 ppb). Soil contained the trace concentrations of other heavy metals, like Pb, Cd and Zn, which made the natural soil background and had no influence on the conducted experiments. Therefore, the soil was classified as uncontaminated.

**Table 1:** Soil and municipal green waste compost properties (mean ± standard deviation *n = 3*)

Parameter	Soil (blank sample)	MGW compost
pH (H2O)	6.45 ± 0.01	8.10 ± 0.02
Organic carbon [g kg^−1^ dry weight]	5.47 ± 0.83	34.46 ± 1.25
Total nitrogen [g kg^−1^ dry weight]	0.52 ± 0.10	1.95 ± 0.09
Available phosphorous [g kg^−1^ dry weight]	0.38 ± 0.07	22.14 ± 1.12
Hg [mg kg^−1^ dry weight]	nd	nd
Pb [mg kg^−1^ dry weight]	0.047 ± 0.005	0.786 ± 0.089
Cu [mg kg^−1^ dry weight]	0.023 ± 0.003	1.352 ± 0.578
Zn [mg kg^−1^ dry weight]	0.029 ± 0.008	0.847 ± 0.706

*nd* not detected

Compost used in the investigations had alkaline pH. This commercially available product was made from green wastes; therefore, the concentration of organic carbon constituted above 30 % of its composition. The concentrations of the chosen heavy metals were low and did not exceed the quality standards for this kind of product. The application of compost to the soil resulted in changing the pH. In all stages of the experiment, the pH of growing media was neutral. This condition can affect the mobility of Hg (Schuster 1991).

### Plant growth and Hg accumulation

#### Effect of Hg in soil on *L. sativum* L

**Fig. 1:**
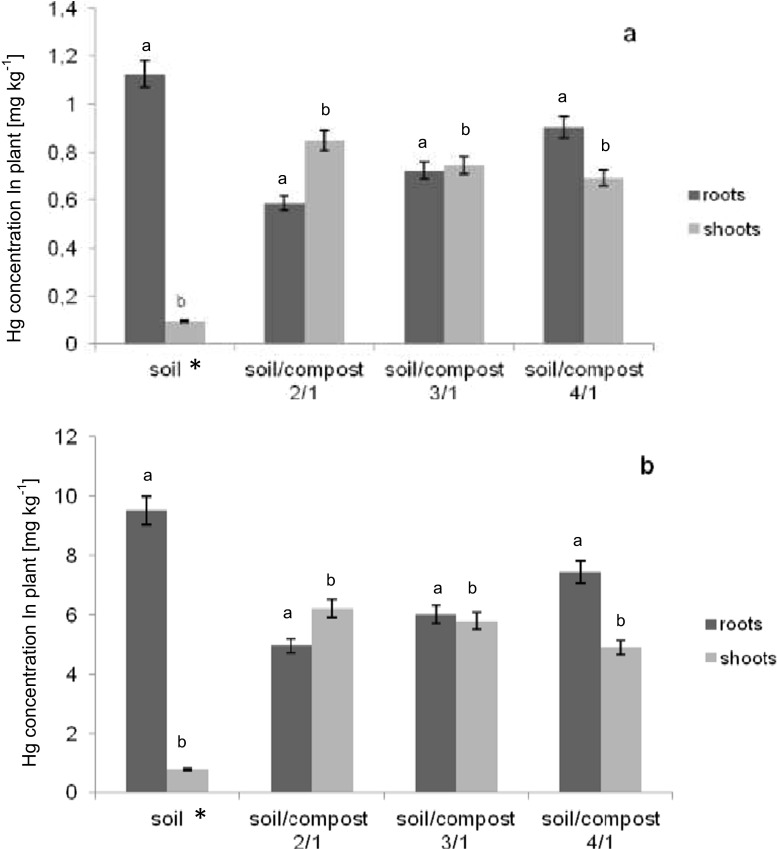
Concentration of Hg in roots and shoots of *Lepidium sativum* L. for soil polluted by **a** 10 mg kg^−1^ soil dry weight of HgCl_2_ and **b** 100 mg kg^−1^ soil dry weight of HgCl_2_

*L. sativum* L. plants were able to accumulate Hg (Fig. 1). For control treatment, over 90 % of Hg was accumulated in roots of plants. The amount of Hg in soil influenced the total accumulation by whole plant. The lowest Hg accumulation by garden cress was observed for soil contamination by 100 mg kg^−1^ soil dry weight of HgCl_2_. This result is a confirmation of investigations conducted by Perez-Sanz et al. (2012) for *Silene vulgaris* L. and Shiyab et al. (2009) for *Bressica juncea* L., who stated that increasing concentration of Hg in soil negatively affect plant accumulation. The tendency of limited Hg translocation to stems and leaves of different plant species was observed by several other authors. For example, Rodriguez et al. (2003) reported that during the Hg phytoextraction by crop plants *Hordeum vulgare* L., *Triticum aestivum* L., and *Lupinus arborelus* L., considerable amount of pollutant was also accumulated in the roots. Slight transfer of Hg to aboveground parts of the plants can be a result of plant response to stress conditions caused by mercury presence in soil. The typical answer is either formation of phytochelatines in plant roots or binding the pollutants to the cell wall of root and as a consequence, deposition of pollutant in that part of the plant (Suszczynski and Shann 1995; Greger et al. 2005).

The stress conditions caused the limitation of *L. sativum* L. biomass compared to soil sample where the cultivation was provided in the unpolluted soil (Table 2.). The lowest plant biomass was obtained for process where 100 mg of Hg per kilogram of soil dry weight was added to the soil. Moreover, visual symptoms of chlorosis and necrosis were also observed. These results agreed with those presented by Perez-Sanz et al. (2012), who reported that plant biomass significantly decreased when concentration of Hg in soil increased. Mercury also influenced the biometric parameters of a plant like lengths of above and below tissues (Table 2.). After 10 mg kg^−1^ soil dry weight Hg amendment, length of shoots and roots of *L. sativum* L. decreased over 27 % and 53 %, respectively, compared to the blank soil sample. According to Patra and Sharma (2000), the effect of Hg on length of roots and shoots of higher plants such as *Brassica oleracea* L., *Chinese cabbage* L., *Beat vulgaris* L., and *Pisum sativum* L., is inversely proportional to the concentration of Hg. Therefore, the Hg occurred to be a very toxic contaminant that influenced the plant biomass, its biometrical parameters, as well as its accumulation and translocation.

**Table 2:** Biomass of *Lepidium sativum* L. and its biometrical parameters. Different letters indicate significant difference at *p*<0.05 by LSD test

Hg concentration added to soil [mg kg^−1^ dry weight]	Growing medium	Plant biomass [g]		Length of plant [cm]	
		Shoots	Roots	Shoots	Roots
0	Soil (blank sample)	18.70^a^ ± 0.37	11.36^a^ ± 0.15	7.54 ± 0.12	4.58 ± 0.15
10	Soil (control sample)	16.63^a^ ± 0.40	6.05^a^ ± 0.07	5.47 ± 0.25	2.14 ± 0.11
	Soil/compost 2/1	20.46^b^ ± 0.22	8.54^b^ ± 0.44	7.21 ± 0.32	3.01 ± 0.02
	Soil/compost 3/1	19.04^b^ ± 0.18	8.08^b^ ± 0.15	6.29 ± 0.20	2.67 ± 0.11
	Soil/compost 4/1	18.47^a^ ± 0.17	6.60^a^ ± 0.22	5.99 ± 0.40	2.14 ± 0.15
100	Soil (control sample)	15.24^a^ ± 0.17	4.05^a^ ± 0.10	4.03 ± 0.09	1.07 ± 0.10
	Soil/compost 2/1	16.70^b^ ± 0.44	7.43^b^ ± 0.14	6.00 ± 0.17	2.67 ± 0.22
	Soil/compost 3/1	14.35^c^ ± 0.16	8.36^c^ ± 0.26	5.20 ± 0.15	3.03 ± 0.14
	Soil/compost 4/1	14.57^a^ ± 0.15	5.83^a^ ± 0.14	5.12 ± 0.15	2.05 ± 0.08

#### Effect of compost amendment on the Hg phytoextraction

Application of compost improved the phytoextraction of mercury-contaminated soil. At this point, it should be stated that all of the comparisons of obtained results include the real concentration of Hg in the growing medium, not the concentration in control soil samples. Amendment of compost to contaminated soil changed the amount of Hg in the growing medium. The higher the compost content, the lower the Hg pollution of growing medium was observed. Therefore, for the variant of experiment where 10 mg kg^−1^ soil dry weight of HgCl_2_ was used as a pollutant, the concentration of Hg in the growing medium (in milligrams per kilogram dry weight) was as follows: for soil/compost ratio 2/1, 3/1, and 4/1—6.623 ± 0.031, 7.482 ± 0.021 and 7.980 ± 0.018, respectively. Similar determinations were carried out for soil pollution by 100 mg kg^−1^ soil dry weight of HgCl_2_. For this part of the investigation, the amount of Hg in the growing medium (in milligrams per kilogram dry weight) for soil/compost ratio 2/1 was 66.457 ± 0.182, for soil/compost ratio 3/1 was 74.262 ± 0.158, and for growing medium where four parts of soil per one part of compost were used was 78.745 ± 1.000.

According to Fig. 1, the Hg concentration in *L. sativum* L. was dependent on both the concentration of pollutant as well as the growing medium composition. For the part of investigations where 10 mg kg^−1^ soil dry weight of HgCl_2_ was used as a soil contaminant (Fig. 1), Hg was accumulated in roots and shoots of *L. sativum* L. plants. Application of compost to contaminated soil increased the total accumulation by plant in all variants of the conducted experiment. The highest total accumulation of Hg was observed for the process carried out in the growing medium consisted of soil and compost in soil/compost ratio 4/1. In this stage, the Hg concentration in plants was higher over 30 % compared to the process provided in the control sample. However, 63 % of total Hg was still accumulated in roots of *L. sativum* L. The satisfactory results were also obtained for the phytoextraction process carried out in the growing medium for soil/compost ratio 3/1. In this variant of investigation, the total accumulation of Hg increased over 20 % compared to the control soil sample. Almost half of the Hg accumulated by the plant was translocated into the shoots. The slight increase of total accumulation was observed for the process provided in the growing medium soil/compost 2/1. Nevertheless, the increase of Hg amount in shoots was observed—63 % of the total mercury concentration was translocated and accumulated in stems and leaves. Increasing transfer of pollutant to aboveground parts of *L. sativum* L. can be explained by both decreasing Hg concentration as well as lowering toxicity in the growing medium. Compost is a product that is characterized by high organic matter content. On the other hand, Hg is known as an element that can easily form complex compounds with different organic molecules. Therefore, the supposition was made that Hg present in the growing medium created complex compounds with organic matter of compost, which resulted in decreasing toxicity of Hg as well as increasing uptaking of pollutant during plant nutrition. In a consequence, the Hg transferred to aboveground parts of *L. sativum* L. was not limited.

Compost obtained from green wastes is often used as a natural fertilizer that changes the soil properties and improves the plant nutrition. In this investigation, compost application to polluted soil had positive effect on the growth of *L. sativum* L. Moreover, the plants cultivated in the growing media with different compost content exhibited no toxicity symptoms during the entire period of cultivation regardless of growing medium composition. For plant cultivation in growing medium consisted of soil polluted by 10 mg kg^−1^ soil dry weight of HgCl_2_ and compost in soil compost ratio 2/1, the plant biomass was similar to what was obtained after plant cultivation in unpolluted blank soil sample (Table 2). The decreasing content of compost in polluted growing medium contributed to decreasing plant biomass. Simultaneously, the length of roots and shoots of plants decreased. Nevertheless, the plant biomass obtained after cultivation in the growing medium—soil/compost 4/1— was over 10 % higher compared to plants cultivated in polluted soil without compost amendment, and the increase of shoot biomass was also observed. These results enabled the statement that *L. sativum* L. showed a high tolerance to mercury during the phytoextraction assisted by compost. Results presented here are controversial to results obtained by Karami et al. (2011), who made the investigations on the efficiency of green waste compost amendment on other heavy metals, like lead and copper mobility and uptake. These authors reported that green waste compost improved the plant development with increased biomass of ryegrass. This is a confirmation of results presented here for growth of *L. sativum* L. However, in the cited experiment, compost application contributed to minimal translocation of pollutants from roots to shoots of ryegrass. In the presented study, the translocation of Hg was dependent on growing medium composition—and for experiments conducted in soil/compost ratio 2/1, the significant increase of Hg translocation to aboveground parts of plants was observed. This could be explained by differences in soil, compost properties, and used plant, compared to materials examined by Karami et al. (2011), as well as by differences in chemical properties and toxicity of Hg, compared to the elements investigated by these authors.

**Fig. 2:**
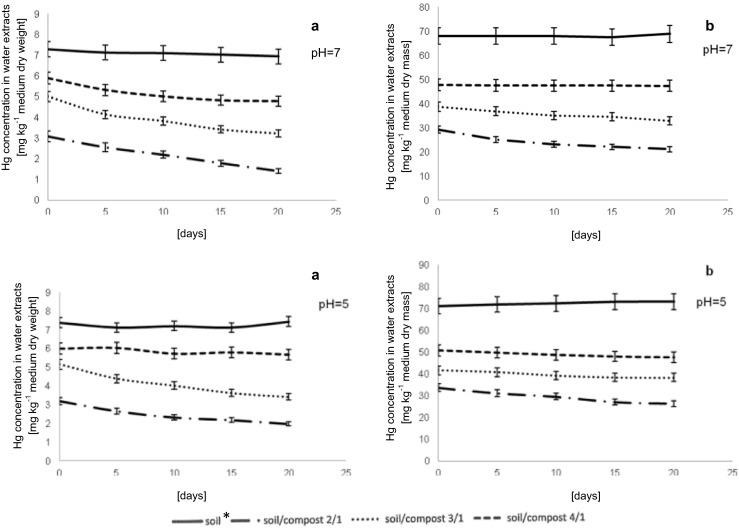
Hg concentration in water extracts (pH = 7 and pH = 5) for mercury pollution. **a** 10 mg kg^−1^ soil dry mass of HgCl_2_. **b** 100 mg kg^−1^ soil dry mass of HgCl_2_

The other part of the experiment consisted of conducting the phytoextraction process enhanced by compost for soil pollution by 100 mg kg^−1^ soil dry weight of HgCl_2_ (Fig. 2). The results of this investigation showed similar tendency to results obtained for lower Hg soil contamination. The highest Hg accumulation by whole plant occurred in the process conducted in the growing medium soil/compost 4/1. Hg was accumulated in roots of *L. sativum* L. Only 33 % of pollutant was transported to the aboveground parts of the plants. The improvement of phytoextraction process was also observed for plant cultivation in soil/compost ratio 3/1. However, in this variant of experiment, Hg transport to shoots was limited, which resulted in higher concentration of Hg in belowground part of a plant. For process conducted in polluted growing medium where two parts of soil per one part of compost was used, Hg was easily translocated to stems and leaves and accumulated. Over 59 % of total Hg was accumulated in shoots of plant.

Similarly, like in previous investigation, compost had positive effect on the plant growth (Table 2). There were no visual symptoms of plant disease observed. Moreover, the compost amendment to polluted soil increased plant biomass regardless of growing medium composition. Mercury presence in growing medium contributed to changes in the biometric parameters of plants. Irrespective of growing medium composition the length of shoots was higher than roots, which resulted in increasing biomass of aboveground parts of the plants. This situation is advised from the phytoextraction’s point of view, because aboveground parts of the plants are easily harvestable.

The application of compost in both variants of conducted experiments—for soil polluted by 10 and 100 mg kg^−1^ soil dry weight of HgCl_2_—increased the total accumulation by *L. sativum* L. This could be caused by the formation of Hg complex compounds with organic matter in soil. New-formed organic mercury compounds are easier to uptake from the soil by plants then inorganic ones as a consequence of different biochemical properties of organic Hg compared to inorganic Hg (Gnamus et al. 2000; Henriques et al. 2013). Henriques et al. (2013) reported in their study that bioaccumulation factor for organic mercury was on average 13 times higher than the respective values for inorganic mercury. Therefore, the larger uptake of Hg can be partially related to higher affinity of plants to mercury organometallic complexes in relation to inorganic Hg. Compost had also positive effect on the translocation of Hg to the aboveground parts of the plants. This result confirmed the conclusion from investigation carried out by Boszke et al. (2008) who observed that availability of Hg in soils for plants was dependent on its chemical form. These authors stated that the availability of organic Hg in soils for roots uptake was higher than that of inorganic mercury. Organic Hg compounds can be easily translocated from roots to aerial parts of the plants (Henriques et al. 2013). According to Krupp et al. (2009), the easier translocation of organic Hg compared to inorganic one occurs because phytochelatines can sequester Hg(II) but not organic Hg, and therefore there is no retention of the organic forms of the metal in the roots as occurs for inorganic Hg.

The efficiency of phytoextraction is determined by two factors: biomass production and bioconcentration factor (McGrath and Zhao 2003). Bioconcentration factor (BCF) was described as the ratio of pollutant accumulated by shoots of *L. sativum* L. to its concentration in the growing medium. Table 3 shows that BCF was dependent on the Hg concentration in soil and growing medium composition. The BCF values obtained for soil contamination by 10 mg kg^−1^ soil dry weight of HgCl_2_ were greater than BCF values for soil contamination tenfold higher. In all variants of investigations, BCF decreased with decreasing compost amount in growing medium. BCF was highest for process conducted in growing medium consisted of soil contaminated by 10 mg kg^−1^ soil dry weight of HgCl_2_ and compost in soil/compost ratio 2/1. In this variant of experiment, BCF increased over one and a half times compared to process conducted in polluted soil. Regardless of pollutant concentration in growing medium, BCF increased in all variants of experiments compared to samples without compost application.

**Table 3:** Bioconcentration factor (BCF) for *Lepidium sativum* L. cultivated in different growing medium

Hg concentration	Soil (control samples)		Growing medium					
			Soil/compost 2/1	Soil/compost 3/1	Soil/compost 4/1	Soil/compost 2/1	Soil/compost 3/1	Soil/compost 4/1
	10	100	10			100		
BCF	0.009	0.008	0.128	0.100	0.120	0.093	0.078	0.062

### Leaching of Hg after phytoextraction process

Leaching of Hg was provided in water extracts in neutral and acidic solutions for examination of Hg mobility after plant cultivation in growing medium enriched by compost. The concentration of Hg (mg kg^−1^ dry weight) after plant cultivation was as follows: for soil contamination by 10 mg of Hg per kg soil dry mass—the concentration in soil was 7.485 ± 0.210, and in growing medium for soil/compost ratio 2/1, 3/1, 4/1 was 3.380 ± 0.063, 5.452 ± 0.46, 6.045 ± 0.607, respectively. For 100 mg kg^−1^ of Hg pollution, the amount of this element in soil was at the level 74.760 ± 1.024 and in growing medium included soil and compost in ratio 2/1, 3/1 and 4/1 was 37.668 ± 1.218, 46.042 ± 2.155 and 54.909 ± 1.478, respectively.

Mercury concentration in water extracts was dependent on the soil/compost ratio, pH of solution used for Hg extraction, as well as the time of the conducted experiment (Fig. 2). The amount of Hg extracted from soil contaminated by 10 mg kg^−1^ soil dry weight of HgCl_2_ in both pH values was the highest compared to other growing media. In this variant, the insignificant decrease of Hg concentration was observed during the time of analysis. The same tendency was observed for soil pollution by Hg in concentration 100 mg kg^−1^ soil dry weight. The decreasing concentration of Hg in extracts during the time of analysis may be explained by the lowering amount of extractable fractions of Hg.

Application of compost to polluted soil decreased the Hg extraction from the contaminated growing media in all variants of conducted investigations. The higher compost content, the lower concentration of extracted Hg was observed, regardless of the treatment. Soil enrichment with compost changed the properties of the growing media due to increasing organic matter content. Organic matter is known to bind trace metals affecting their solubility, mobility, and toxicity (Ravichandran 2004; Gray and McLaren 2006). Especially humic substances, which constitute a major part of the organic matter of compost, it can reduce metal solubility by formation of stable metal chelates (Walker et al. 2004). Humic compounds bear negatively charged sites on carboxyl and phenol groups, allowing metal complexation. Metal can be precipitated, adsorbed on organic matter, or can be in a soil solution as soluble organic complexes (Stevenson 1982).

The differences in concentration of Hg extracted from soil/growing media were dependent on the pH of the solution used for extraction. Solution with acidic pH affected Hg extraction from all contaminated growing media. For growing medium consisted of soil and compost in ratio 4/1, the Hg concentration in water extracts was almost at the same level regardless of time of analysis. The highest decrease of Hg amount in water extracts was observed for soil/compost ratio 3/1 and was over 33 % lower at the end of the experiment compared to its first determination. However, the Hg concentration in this variant at pH = 5 increased compared to values obtained at neutral pH. Insignificant decrease of Hg amount was observed for soil/compost ratio 2/1.

The similar results were observed for experiments conducted after soil contamination by 100 mg kg^−1^ soil dry weight of Hg (Fig. 2). The amount of Hg in water extracts was lower from 15 to 27 % depending on the compost content in growing medium relative to polluted soil (blank soil sample). After comparison of the results obtained for both solutions used for extraction, the Hg concentration was lower in water extracts at neutral pH regardless of growing medium composition, than in acidic one. These results are consistent with the researches conducted by Francoise et al. (2004), who stated that the concentration of other heavy metals like Zn, Cd, and Pb in water extracts was lower when compared to extraction in acidic solution. The higher amount of Hg extracted from contaminated soil or growing medium in acidic pH is dependent on the fraction of Hg that can be extracted. According to Rodrigues et al. (2010), using HNO_3_ solution for Hg extraction leads to extract not only bioavailable forms of Hg but also its reactive fractions, which include Hg^2+^, Hg(OH)_2_, HgCl_2_, HgBr_2_, and Hg^2+^ complexes with organic acids and methylmercury. Moreover, in acidic pH, the weaker Hg complexes with organic matter can be broken down and/or Hg partially associated to amorphous oxides can be dissolved. Therefore, Hg concentration obtained after extraction in acidic pH was higher compared to neutral solution.

## Conclusions

This study has found that *L. sativum* L. has the capacity to accumulate Hg from contaminated soil. Hg accumulation was dependent on soil treatment and correlated with plant biomass and the biometric plants parameters. Potential leaching of Hg after phytoextraction was based on the Hg extraction in acidic and neutral solutions.

Mercury has found to be a toxic element that limits the efficiency of phytoextraction process due to its low plant accumulation and translocation to aerial tissues. Hg has also negatively influenced the plant biomass and the length of both roots and shoots of *L. sativum* L. Application of compost as an amendment during assisted phytoextraction of soil contaminated by Hg has a positive impact on the efficiency of the process. Also, plants’ biomass and their biometric parameters were positively affected. Regardless of the treatment, after compost incorporation to polluted soil, the total accumulation of Hg increased. Compost amendment facilitated the translocation of pollutant to shoots. The higher compost content in growing medium, the higher translocation of Hg occurred. Compost also stimulated the plant growth through the increase of *L. sativum* L. biomass, especially its aboveground parts.

Phytoextraction of Hg-contaminated soils enhanced by compost with the use of *L. sativum* L. can be recommended not only for the increasing effectiveness of the process and positive influence on the biomass increase but also due to the low extraction of Hg after the treatments. Compost amendment decreased Hg concentration extracted from growing media in both neutral and acidic solutions, compared to the process without compost amendment. The higher amount of Hg was extracted in acidic solution relative to neutral one as a result of extraction of reactive and available fractions of Hg from soil.

In conclusion, *L. sativum* L with soil enrichment using compost can be recommended for the phytoextraction of Hg from polluted soil.
